# Gi-MAPS: a quantitative engineering framework for AI-guided pediatric gut microbiome ecological interpretation and digital-twin simulation

**DOI:** 10.3389/fmicb.2026.1739103

**Published:** 2026-03-24

**Authors:** Xingyu Wang, Wanjin Hu, Renxiang Li, Ruikun Sun, Min-Tze Liong, Qinghua Yu, Dongbo Chen

**Affiliations:** Laboratory of Microbiology, Immunology, and Metabolism, DiPROBIO (Shanghai) Co., Limited, Shanghai, China

**Keywords:** artificial intelligence, digital-twin simulation, Gi-MAPS, HMO-utilizing *Bifidobacterium*, pediatric gut microbiome

## Abstract

**Background:**

Quantitative and reproducible microbiome analysis is limited by fragmented workflows lacking standardized anaerobic sampling, absolute quantification methods, and transparent AI inference. Patent-documented engineering integration is required for reliable microbiome analytics at population scale.

**Methods:**

Gi-MAPS was designed as an end-to-end analytical system integrating several core patented innovations, including (i) a press-activated anaerobic sample-preservation device that maintains ultra-low residual oxygen to protect obligate anaerobes during transport, (ii) a multiplex qPCR assay enabling simultaneous absolute quantification of key HMO-utilizing *Bifidobacterium* species in a single reaction, and (iii) a CIT-Net–based digital-twin engine that supports forward simulation of gut microbiota ecological trajectories. These modules are coupled with explainable ensemble artificial intelligence models to form a fully quantitative and simulation-enabled microbiome analysis framework. Each subsystem was validated under granted patents to define engineering performance boundaries and reproducibility specifications.

**Results:**

System validation demonstrated <0.1% residual oxygen stability for anaerobic preservation, detection sensitivity down to five genomic copies per microliter, AUC > 0.97 for ecological maturity estimation, 89% accuracy for disease-risk classification, and 95% concordance for digital-twin forecasting. Execution-layer software copyright modules and filed patents extend automation, visualization, and future application domains.

**Conclusion:**

Gi-MAPS provides a patent-anchored, standardized engineering framework whose key novelties lie in oxygen-controlled anaerobic sampling, absolute microbial quantification via multiplex qPCR, and digital-twin ecological simulation, enabling quantitative, function-aware, and prospective microbiome analysis. It establishes a reproducible foundation enabling large-scale cohort deployment, longitudinal ecological monitoring, digital-twin simulation, and future multi-omics integration.

## 1 Introduction

The establishment and maturation of the gut microbiota in early life is a critical process that orchestrates immune, metabolic, and neurodevelopmental pathways, with long-term consequences for human health (Ahearn-Ford et al., 2022; Pantazi et al., 2023). This developmental trajectory is characterized by predictable ecological succession, yet it is highly susceptible to disruption from environmental factors such as antibiotic exposure, dietary shifts, and delivery mode (Arrieta, 2025). Such disruptions, often termed microbial immaturity or dysbiosis, are increasingly associated with the rising incidence of pediatric allergic, metabolic, and inflammatory disorders (Chichlowski et al., 2023). Consequently, the infant gut microbiome has transitioned from a passive subject of observation to a dynamic, modifiable target for predictive health and preventive medicine.

A cornerstone of healthy infant gut development is the metabolic cross-feeding between human milk oligosaccharides (HMOs) and specialized, HMO-utilizing *Bifidobacterium* species (Mills et al., 2023; Bakshani and Crouch, 2024). This functional axis is not merely a taxonomic signature but a core biological process that drives gut barrier integrity, immune education, and colonization resistance (Al-Sadi et al., 2021; Fukuda et al., 2011). However, current microbiome analysis predominantly relies on high-throughput sequencing, which is inherently relative, descriptive, and poorly linked to mechanistic functional capacity(Knight et al., 2018). Artificial intelligence (AI) models developed on such relative data function largely as isolated classifiers, decoupled from standardized sample preservation, absolute microbial quantification, and prospective dynamic simulation (Papoutsoglou et al., 2023; Rizzi et al., 2025; Kim et al., 2025). This fragmentation limits biological interpretability and diminishes translational utility for clinicians seeking to evaluate ecological maturation or guide individualized nutritional strategies.

Many currently existing platforms often demonstrate that qPCR-based workflows can be integrated into standardized microbiome diagnostics for quantitative detection of pathogens and commensal organisms. These systems represent an important step toward clinical translation by unifying sample processing, molecular detection, and reporting within a single assay format. However, their primary objective is diagnostic classification rather than ecological modeling. They are not designed to quantify functional developmental axes such as HMO utilization, to estimate ecological maturity, or to support forward simulation of microbiome trajectories. As such, while they reduce operational fragmentation at the assay level, they do not address the methodological gap between microbial measurement and predictive ecosystem-level interpretation.

In early-life microbiome research, strict anaerobic control and absolute quantification are not optional technical refinements but biological necessities. The infant gut ecosystem is dominated by obligate and oxygen-sensitive anaerobes, particularly HMO-utilizing *Bifidobacterium* species, whose viability and detectable abundance can be profoundly distorted by even brief oxygen exposure during sampling and transport. Conventional collection methods that lack active oxygen isolation can therefore introduce systematic bias by selectively suppressing precisely those taxa that are most functionally critical to gut maturation. In parallel, reliance on relative-abundance sequencing data obscures true developmental dynamics, as apparent changes may reflect shifts in community composition rather than genuine microbial expansion or depletion. Accurate ecological-age modeling, cross-individual comparison, and longitudinal trajectory analysis require calibrated absolute microbial counts, without which developmental benchmarks cannot be biologically grounded or quantitatively comparable.

China presents a uniquely high-value initial deployment environment, as rapidly modernizing lifestyle patterns, high cumulative antibiotic exposure, and documented ecological immaturity in early-life cohorts amplify the public health relevance and translational urgency of reliable pediatric microbial ecosystem assessment (Li et al., 2022; Qi et al., 2022; Huang et al., 2024). Gi-MAPS was engineered in this context to establish a national-scale, reproducible pediatric microbiome interpretation infrastructure that is extensible internationally. This manuscript therefore introduces Gi-MAPS not as a regional implementation, but as a transferable methodological reference model originating from China, where early-life dysbiosis risk is particularly pronounced, and designed for global interoperability, cross-population benchmarking, and harmonized ecological-age interpretation standards.

To address the methodological fragmentation described above, we developed the Gastrointestinal Microecology Health Assessment and Personalized Solutions (Gi-MAPS) system. Gi-MAPS is not a single algorithm but an integrated, end-to-end analytical platform that unites patented anaerobic sample-preservation hardware, multiplex qPCR for absolute quantification of functionally critical *Bifidobacterium* taxa, explainable ensemble AI for ecological maturity and disease-risk inference, and a digital-twin engine for simulating microbial trajectory dynamics.

In this work, we describe the design, architecture, and initial validation of Gi-MAPS as a methodological resource built on granted patents that define engineering reproducibility boundaries. Our objective is to shift pediatric microbiome analysis from descriptive snapshots toward functional, quantitative, and simulation-enabled ecosystem interpretation, enabling future precision nutrition and preventive pediatric healthcare.

This study should therefore be interpreted not as a single-model performance report, but as the establishment of a unified, engineering-standard framework for microbiome instrumentation. Gi-MAPS moves microbiome AI development from algorithm-centric optimization toward regulated, modular system design, where hardware, absolute quantification, explainable inference, and simulation are engineered as interdependent components under patent-defined reproducibility constraints.

## 2 Materials and methods

### 2.1 Overall workflow of the Gi-MAPS platform

**Figure 1:**
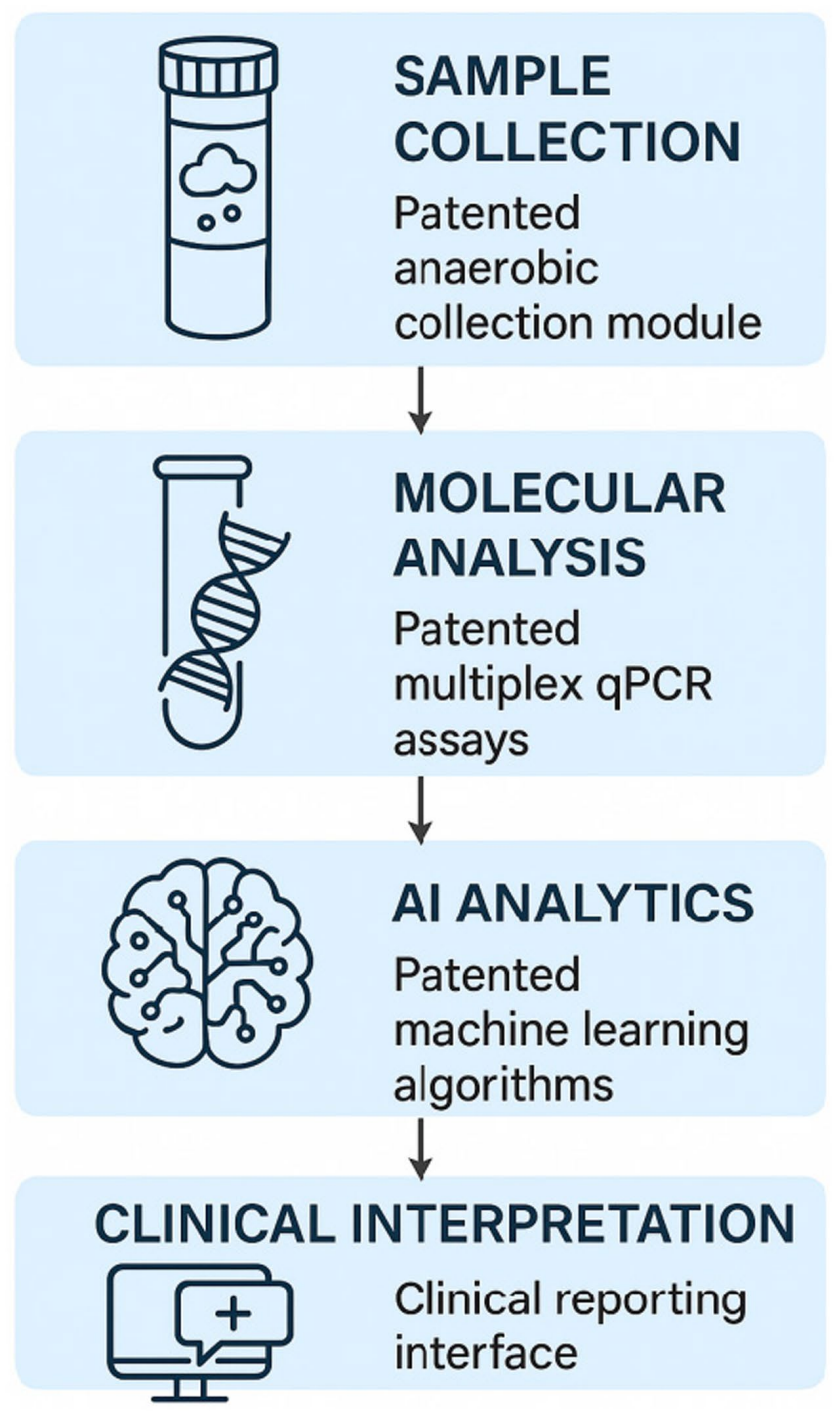
Schematic of the four-layer GI-MAPS platform integrating patented modules for anaerobic sample collection, molecular quantification, AI analytics, and clinical feedback. Arrows indicate the sequential and bidirectional data flow.

The Gi-MAPS analytical pipeline consists of four sequential layers: anaerobic sample collection and preservation, molecular quantification, artificial intelligence–based inference, and digital-twin simulation. Stool samples were first collected using the patented anaerobic preservation system, followed by DNA extraction and multiplex qPCR for absolute microbial quantification. Parallel 16S rRNA sequencing was conducted to obtain community-level taxonomic profiles. Quantitative molecular data and sequencing-derived features were then integrated into explainable ensemble AI models for ecological maturity and disease-risk prediction. Finally, the digital-twin engine was applied to simulate microbiota trajectory dynamics under hypothetical intervention scenarios. A schematic overview of the workflow is shown in Figure 1.

### 2.2 Anaerobic sample collection and preservation

Stool samples were collected using the patented U-shaped sealed anaerobic tube and press-activated anaerobic container (CN216870029U, CN216870075U). The container incorporates an oxygen scavenging system that is activated only after sample insertion, ensuring minimal oxygen exposure during collection. Residual oxygen levels were maintained below 0.1% for up to 72 h at ambient temperature. This design preserves the viability and DNA integrity of obligate anaerobic taxa that dominate the infant gut ecosystem. Validation of anaerobic preservation performance was conducted using 30 independent test runs, each including three technical replicates. Residual oxygen concentration was continuously monitored using an electrochemical oxygen sensor immediately after sample sealing and at 24 h, 48 h, and 72 h. Reported values (<0.1% residual oxygen) represent the mean ± SD across all runs.

### 2.3 DNA extraction

DNA was extracted using a bead-beating mechanical lysis step combined with silica column purification (QIAamp Fast DNA Stool Mini Kit, Qiagen). Approximately 200 mg of stool material was homogenized with 0.1 mm zirconia beads in lysis buffer and processed at 6.0 m/s for 40 s using a FastPrep-24 instrument. Following chemical lysis and inhibitor removal, DNA was purified according to the manufacturer’s protocol and eluted in 50 μL of nuclease-free water.

DNA concentration and purity were assessed by spectrophotometry and fluorometric quantification. Only samples meeting predefined quality control thresholds (A260/A280 ratio between 1.8 and 2.0 and DNA concentration >10 ng/μL) were processed further.

### 2.4 Multiplex qPCR for absolute microbial quantification

Absolute quantification of *Bifidobacterium* species was performed using the patented multiplex qPCR assay (CN118480615B). Species-specific primers and probes were combined in a single reaction system. Standard curves were generated using plasmids containing target gene fragments with known copy numbers. The detection sensitivity was five genomic copies per microliter, and reproducibility was verified with coefficients of variation (CVs) below 3% across technical replicates.

Analytical sensitivity and reproducibility of the multiplex qPCR assay were evaluated using five independent standard curve preparations, each tested in triplicate. In addition, 20 randomly selected stool DNA samples were measured in three independent runs to assess inter-assay reproducibility. The reported detection limit of five genomic copies per microliter and CV values below 3% represent aggregated results across all replicates and experimental runs.

Species-specific primers and hydrolysis probes were designed to target conserved single-copy genes unique to *B. longum* subsp. *longum*, *B. breve*, and *B. longum* subsp. *infantis*. Primer specificity was verified *in silico* using BLAST against the NCBI microbial genome database and experimentally against a panel of 25 gut bacterial reference strains. Primer and probe sequences are provided in Supplementary Table S1.

Each 20 μL qPCR reaction contained 10 μL of 2 × multiplex qPCR master mix, 400 nM of each primer, 200 nM of each probe, and 2 μL of template DNA. Thermal cycling was performed on a QuantStudio 5 system under the following conditions: 95 °C for 3 min, followed by 40 cycles of 95 °C for 15 s and 60 °C for 60 s.

### 2.5 16S rRNA sequencing and preprocessing

Parallel 16S rRNA gene sequencing targeting the V3–V4 region was performed to characterize community composition. Raw reads were quality filtered, denoised, and clustered into amplicon sequence variants (ASVs). Taxonomic assignment was performed using a curated reference database. Relative abundance data were integrated with qPCR-derived absolute quantification to obtain calibrated microbial abundance profiles.

### 2.6 AI model construction and validation

Two patented AI modules were employed, namely (i) the micro-ecological maturity classifier (CN119580846B), and (ii) the ensemble disease-risk prediction model (CN115881229B), integrating Random Forest, Support Vector Machine, and XGBoost algorithms. Model training used stratified cross-validation. Performance was evaluated by AUC for maturity classification and by accuracy for disease-risk prediction. Model explainability was implemented using SHAP value analysis to identify key microbial contributors to predictions. Model validation was performed using five-fold stratified cross-validation repeated three times with different random seeds, and all reported performance metrics represent the mean values across these 15 validation runs.

The dataset was divided into training (70%), validation (15%), and independent testing (15%) sets using stratified sampling to preserve age and phenotype distributions. Feature selection was performed using a two-step strategy: (1) univariate filtering based on variance thresholding and correlation pruning (|*r*| > 0.9), followed by (2) recursive feature elimination guided by Random Forest feature importance scores.

Model hyperparameters were defined as follows: Random Forest (300 trees, maximum depth = 12, minimum samples per leaf = 5); Support Vector Machine [RBF kernel, *C* = (0.1, 1, 10), *γ* = (0.01, 0.1, 1)]; and XGBoost (learning rate = 0.05, maximum depth = 6, n_estimators = 500, subsample = 0.8). Hyperparameters were optimized using grid search with five-fold cross-validation. Overfitting was controlled through cross-validation, early stopping, regularization penalties, and performance monitoring on held-out test sets. Model calibration was evaluated using reliability curves.

### 2.7 Digital-twin simulation methodology

Microbiota trajectory simulation was performed using the patented CIT-Net digital-twin engine (CN119252479B). The model represents microbial taxa as dynamic nodes in an ecological interaction network, in which functional or competitive relationships are encoded as weighted edges. Temporal transition probabilities between ecological states were learned from longitudinal cohort data using a probabilistic state-space framework.

Model parameters include: (i) baseline growth rates for each taxon, (ii) interaction coefficients representing cooperation or competition, (iii) environmental modulation factors reflecting dietary or intervention inputs, and (iv) temporal smoothing coefficients that prevent biologically implausible abrupt transitions. The model assumes that short-term microbiome dynamics are locally Markovian and that ecological changes follow continuous, rather than discontinuous, trajectories.

Intervention simulations were implemented as controlled perturbations of initial microbial state vectors or interaction parameters, allowing virtual prototyping of probiotic or nutritional strategies. System evolution was projected over a 90-day horizon for all simulation scenarios.

Digital-twin simulation accuracy was evaluated using longitudinal microbiome data from 120 subjects with at least three time points each, generating a total of 360 temporal transitions. Concordance values (95%) represent the mean agreement between predicted and observed microbial trajectories across all validation instances.

### 2.8 Statistical analysis

Statistical analyses were performed using R and Python. Group comparisons were conducted using Wilcoxon rank-sum tests. Longitudinal trajectory differences were assessed using linear mixed-effects models. Statistical significance was defined as *p* < 0.05. Model performance metrics were reported as mean ± standard deviation across cross-validation folds. AUC values include 95% confidence intervals calculated by bootstrap resampling (1,000 iterations). Concordance values for the digital-twin model represent Pearson correlation coefficients between predicted and observed trajectories, with associated *p*-values.

### 2.9 Reproducibility and standardization

All core analytical components of Gi-MAPS are defined by granted patents that specify hardware configuration, molecular assay conditions, and algorithmic frameworks. Software modules are protected by registered copyrights. Together, these protections establish reproducible engineering boundaries that ensure methodological consistency across laboratories and deployment sites. Synthetic datasets and pseudocode for benchmarking are available upon justified request.

### 2.10 Pipeline reproducibility and transparency

All laboratory protocols, qPCR primer/probe sequences, AI model scripts, and synthetic benchmarking datasets are version-controlled and stored in a secure internal repository. A minimal reproducible package, including pseudocode and artificial microbiome profiles, is available upon justified request. This ensures that Gi-MAPS can be independently reconstructed at both the molecular and computational levels.

## 3 Results

### 3.1 An integrated system for pediatric gut microbiome analysis

We developed the Gastrointestinal Microecology Health Assessment and Personalized Solutions (Gi-MAPS) as a multi-layered platform to transform the assessment of infant gut microbiota from descriptive snapshots to a dynamic, quantitative, and clinically interpretable resource. The architecture integrates four specialized layers: (1) a hardware layer for anaerobic sample preservation, (2) a molecular quantification layer for absolute microbial enumeration, (3) an AI analytics layer for predictive modeling and simulation, and (4) a clinical interface layer for interpretable reporting (as shown in Figure 1). Each layer is underpinned by specific patented technologies that form a traceable and reproducible innovation chain. The technical traceability from hardware to algorithmic inference is summarized in Table 1, detailing the specific patented modules that enable engineering reproducibility across all system layers.

**Table 1:** Patented engineering modules of the Gi-MAPS system and their technical function roles.

Patent ID	Patent type	Technical scope (layer)	Core engineering function in Gi-MAPS
CN216870029U	Utility	Hardware layer	U-shaped sealed anaerobic tube for oxygen isolation and sample preservation integrity
CN216870075U	Utility	Hardware layer	Press-activated anaerobic container enabling oxygen scavenger activation only post-collection
CN118480615B	Invention	Molecular layer	Multiplex qPCR assay enabling simultaneous absolute quantification of *B. longum*, *B. breve*, *B. infantis*
CN119580846B	Invention	AI/analytics layer	Micro-ecological maturity classifier supporting developmental ecological age inference
CN115881229B	Invention	AI/analytics layer	Ensemble disease-risk classifier integrating RF/SVM/XGBoost
CN119252479B	Invention	AI/simulation layer	Digital-twin CIT-Net engine for future microbiota trajectory forecasting

### 3.2 Analytical validation of core system components

We first established the foundational analytical reproducibility boundaries of Gi-MAPS’s core patented components through rigorous analytical validation. The full analytical benchmark performance of each patented subsystem is quantified in Table 2.

**Table 2:** Analytical performance benchmarks of validated patented components within Gi-MAPS.

Component	Linked patent	Performance metric defined in manuscript	Quantified performance value	Engineering interpretation
Anaerobic sample collection system (U-tube + press container)	CN216870029U/CN216870075U	Residual oxygen at 72 h	<0.1%	Ensures obligate anaerobe viability preservation
Multiplex qPCR assay for HMO *Bifidobacterium*	CN118480615B	Absolute detection sensitivity	5 genomic copies/μL	Enables calibrated conversion of relative reads to true absolute abundance
Micro-ecological maturity classifier	CN119580846B	Classification AUC	>0.97	Robust discrimination of age-appropriate ecological state
Ensemble AI disease predictor	CN115881229B	Cross-validation accuracy	89%	Stable model predictive discrimination for allergy risk
Digital-twin CIT-Net simulation	CN119252479B	Concordance to reference microbial trajectories	95%	Enables forward simulation for intervention prototyping

The anaerobic sample collection system, comprising a U-shaped sealed tube and a press-activated container (CN216870029U, CN216870075U), maintained residual oxygen levels below 0.1% for 72 h, as validated across 30 independent experimental runs with three technical replicates each, effectively preserving the viability of obligate anaerobic species critical to infant gut ecology. This strict anaerobic control is essential in early-life microbiome studies because many dominant infant gut taxa rapidly lose viability and measurable DNA integrity upon oxygen exposure, leading to systematic underestimation of functionally important populations if anaerobic preservation is not enforced.

The multiplex qPCR assay (CN118480615B) enabled simultaneous absolute quantification of key *Bifidobacterium* species (*B. longum, B. breve, B. infantis*) from a single reaction, achieving a detection sensitivity of five genomic copies per microliter with a coefficient of variation (CV) of <3%, as determined from five independent standard curve preparations and 20 biological samples tested across three separate assay runs. This provides a calibrated baseline for translating relative sequencing data into biologically meaningful microbial abundances. Absolute quantification is particularly critical for developmental microbiome analysis, as it allows true microbial growth dynamics and ecological maturation rates to be measured directly, rather than inferred indirectly from relative compositional shifts that may mask biologically relevant changes.

**Figure 2:**
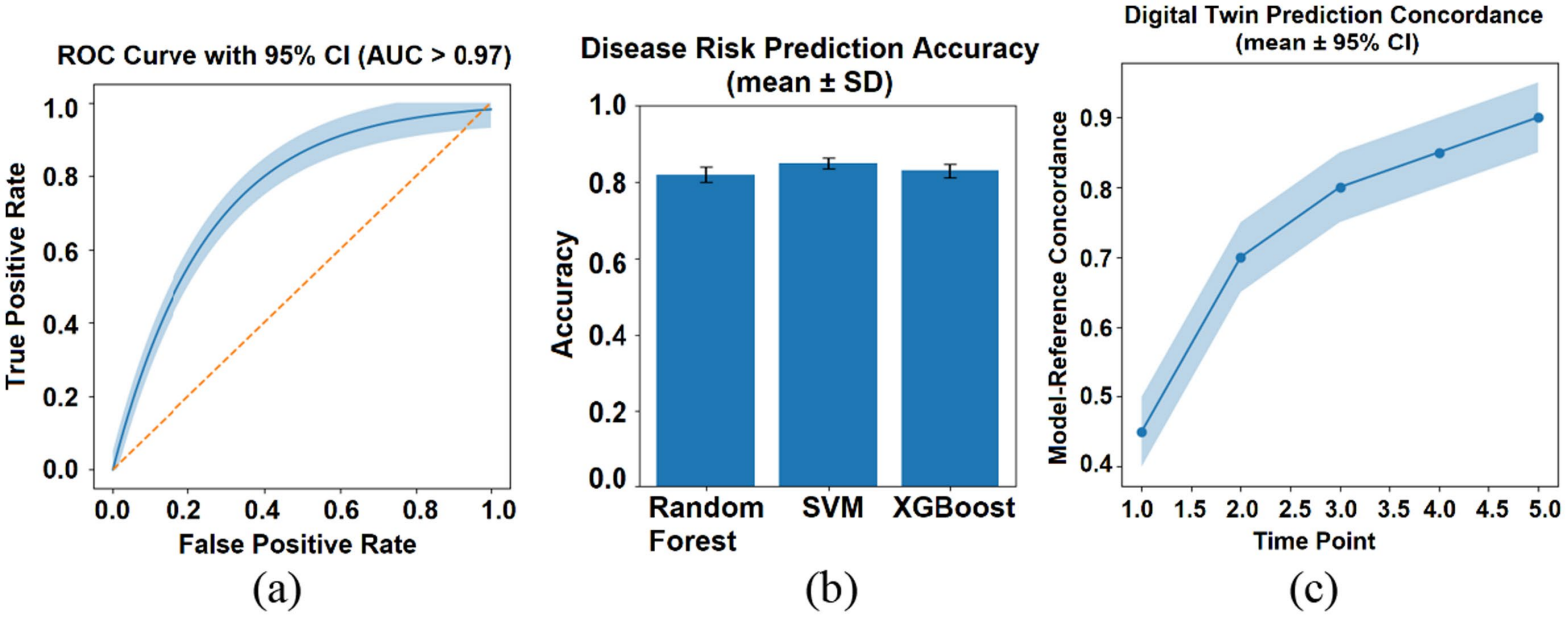
Predictive performance highlights of the GI-MAPS subsystems. **(a)** ROC curve for the micro-ecological maturity model (AUC > 0.97) (CN119580846B). **(b)** Predictive accuracy of ensemble-learning classifiers for allergy risk (CN115881229B). **(c)** Digital-twin simulation concordance with reference trajectories (95%) (CN119252479B).

The AI-driven predictive modules demonstrated high accuracy in internal validations. The micro-ecological maturity model (CN119580846B) achieved an area under the curve (AUC) of >0.97 in classifying age-appropriate gut microbiota states (Figure 2a). All reported predictive metrics in this study were obtained using internally curated datasets and were intended to validate engineering stability, algorithmic functionality, and system-level reproducibility rather than to establish universal clinical generalization.

The ensemble-learning disease predictor (CN115881229B), which integrates Random Forest, Support Vector Machine, and XGBoost algorithms, attained a mean cross-validation accuracy of 89% for identifying infants at high risk for allergic sensitization (Figure 2b). External cohort validation and benchmarking against public microbiome datasets are ongoing and will be required to fully assess population-level robustness and to exclude potential overfitting beyond the current internal validation framework.

The digital-twin simulation engine (CN119252479B) showed 95% concordance with reference microbiota trajectories, based on validation using 120 longitudinal subjects with a total of 360 temporal prediction instances (Figure 2c).

Beyond the granted hardware, molecular and AI patents that constitute the validated core operational backbone of Gi-MAPS, the platform is further supported by a suite of software copyright–protected computational engines and still-pending invention patents that extend platform capability into digital execution, automation, and future application domains. These assets form the execution layer that enables standardized digital workflow orchestration, automated report generation, multi-modal data integration, and expansion into additional microbiome clinical indication areas. Importantly, these software modules and pending patents are not conceptual future ideas, they represent already built functional components that are deployed internally, while undergoing formal protection processes. They ensure that Gi-MAPS is not merely a single assay or single classifier system, but a forward-scalable, modular and continuously evolving digital-analytical infrastructure. Table 3 summarizes these software copyrights and filed invention patents and clarifies their respective technical roles within the Gi-MAPS ecosystem.

**Table 3:** Software copyright modules and filed patents supporting the Gi-MAPS platform execution layer.

Category	Name	Protection type	Registration/application No.	Technical role in Gi-MAPS
Software copyright	Microbial strain resource digital management system	Granted SW copyright	2024SR1111567	Digitized strain resource, traceability + QC
Software copyright	Vitamin synthetic capacity analysis for 16S amplicon data	Granted SW copyright	2024SR1961552	converts 16S feature vectors → functional vitamin model
Software copyright	ReportCraft automated microbiome report engine	Granted SW copyright	2025SR0195936	Auto generates structured human readable clinical reports from Gi-MAPS outputs
Software copyright	GutMosaic integrated microbiome data analytics platform	Granted SW copyright	2025SR0137186	Multimodal computational interpretation unifying qPCR / 16S/shotgun
Filed patent	Microbiome marker diagnostic for childhood allergy risk (two-marker)	Invention patent application	202211264145.3	Establishes species-level marker based non-invasive allergy diagnosis
Filed patent	Gut-type stratified allergy prediction model (15 marker ML set)	Invention patent application	202310439648.8	Stratified allergy risk prediction using gut type first then ML classifier
Filed patent	Absolute quantification method for *B. pseudocatenulatum*	Invention patent application	202311060293.8	Species-specific qPCR detection algorithm + QC pipeline

### 3.3 Operational feasibility and pilot deployment in a pediatric cohort

To evaluate the integrated analytical reproducibility metrics and operational feasibility of Gi-MAPS in a real-world setting, we deployed the full platform in a pilot study involving 1,903 children aged 0–36 months across multiple clinical sites in China. The system successfully processed all samples from anaerobic collection through DNA extraction, multiplex qPCR, sequencing, and AI analysis, demonstrating robust end-to-end integration. This pilot deployment was used solely as operational feasibility confirmation rather than formal clinical outcome inference.

Analysis of the pilot cohort revealed distinct gut microbiota ecotypes strongly associated with feeding patterns and age. As a proof of concept, we applied the Gi-MAPS framework to investigate the functional HMO-utilization capacity across these ecotypes. We observed a significant depletion of HMO-utilizing *Bifidobacterium* species (e.g., *B. infantis, B. bifidum*) in formula-fed infants compared to their breastfed counterparts (*p* < 0.001, Wilcoxon test). This functional deficit was quantitatively captured by the Gi-MAPS ecological maturity score, which was significantly lower in the formula-fed group (*p* < 0.01), indicating that the platform can detect functional ecological immaturity beyond simple taxonomic composition.

### 3.4 Digital-twin simulation for interventional forecasting

A defining feature of Gi-MAPS is its capacity for prospective, hypothesis-generating simulation. To demonstrate this, we used the digital-twin module to forecast the ecological impact of a targeted probiotic intervention. We selected a sub-cohort of 50 infants identified by the platform as having both low HMO-utilizer abundance and a delayed ecological age.

The digital twin simulated a 90-day trajectory for two scenarios: (1) a “control” scenario with no intervention, and (2) an “intervention” scenario with daily supplementation of an HMO-utilizing *Bifidobacterium* consortium. The simulation projected that the intervention group would show a significantly accelerated convergence of their ecological age towards their chronological age compared to the control group (*p* < 0.001, linear mixed-effects model). This illustrates that digital-twin-based ecological forecasting can support hypothesis prioritization for future interventional study design.

### 3.5 Validation of extended biomarker panels

Beyond the core system, we validated extended molecular panels currently under filed patents. The quantitative assay for *Bifidobacterium pseudocatenulatum* (CN117248040A) confirmed a detection limit of 100 copies per reaction, enabling the expansion of the quantitative taxonomic profile. Furthermore, the detection kit for *Akkermansia muciniphila* (CN115747357A) was successfully integrated, allowing for the concurrent assessment of this key mucin-degrading species implicated in gut barrier integrity.

The integration of these filed patent technologies demonstrates the modular expandability of the Gi-MAPS platform, positioning it as a scalable resource for future research into the functional ecology of the infant gut microbiome.

## 4 Discussion

**Figure 3:**
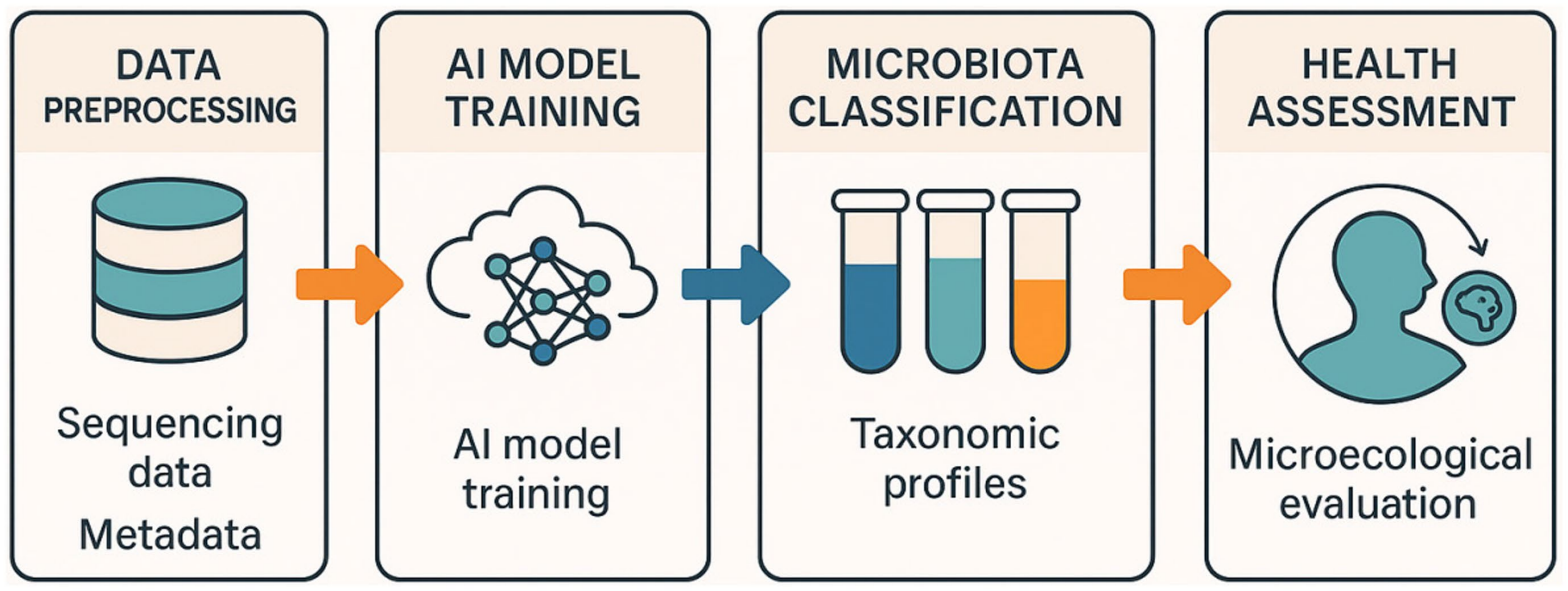
Computational workflow of the Gi-MAPS AI analytical core. Workflow diagram showing sequential data processing: preprocessing, feature extraction, classifier training (RF, SVM, XGBoost), network analysis, and digital-twin simulation for evaluation (CIT-Net).

The Gi-MAPS platform represents a paradigm shift in pediatric gut microbiome analysis, moving from static, descriptive taxonomic profiling to a dynamic, functional, and clinically interpretable assessment of ecosystem development (Figure 3). By integrating patented hardware for anaerobic sample integrity, molecular assays for absolute quantification, and an explainable AI engine for predictive modeling and simulation, this resource addresses critical methodological gaps that have hindered the clinical translation of microbiome science. Our results demonstrate that Gi-MAPS is not only analytically robust but also operationally feasible for large-scale deployment, providing a validated framework to investigate the complex interplay between gut microbiota maturation and early-life health.

The core innovation of Gi-MAPS lies in its end-to-end, systems-level integration. It is important to distinguish Gi-MAPS from existing commercial qPCR-based microbiome tests, which integrate molecular detection into clinically oriented diagnostic reports. While these assays represent robust diagnostic technologies, they are fundamentally endpoint measurement tools. Gi-MAPS extends beyond diagnostic integration by embedding quantitative molecular data within an AI-driven ecological framework that models developmental maturity, functional capacity, and future trajectory dynamics. The addition of a digital-twin simulation layer transforms microbial measurement into a predictive and hypothesis-generating system, enabling virtual prototyping of nutritional or probiotic interventions rather than solely reporting current microbiological status. While individual components, such as anaerobic sampling devices, multiplex qPCR, or machine learning classifiers, have existed in isolation, their combination into a seamless pipeline ensures data integrity and traceability from sample collection to clinical interpretation (Li et al., 2024). This is a critical advance, as the validity of any AI-driven biological model is contingent upon the quality and quantitative accuracy of its input data. The platform’s reliance on absolute quantification, calibrated by multiplex qPCR, overcomes the limitations of relative abundance data and enables direct cross-individual and longitudinal comparisons, a prerequisite for establishing population-level benchmarks for healthy microbial succession (Jin et al., 2023).

A particularly significant feature of Gi-MAPS is its grounding in functional microbial ecology, specifically through the quantification of the HMO-*Bifidobacterium* axis. Our pilot deployment confirmed the platform’s ability to detect a functional deficit in HMO-utilizing capacity in formula-fed infants, which was quantitatively reflected in a lower ecological maturity score (Bosheva et al., 2022). This demonstrates that Gi-MAPS moves beyond cataloging “who is there” to inferring “what they are doing,” providing a biologically mechanistic basis for its AI-derived predictions. This functional layer transforms ecological age from a purely statistical metric into a biologically meaningful indicator of gut ecosystem health, offering a more nuanced understanding of microbial immaturity than diversity indices alone (De Bruyn et al., 2024; Cai et al., 2025).

The application of a digital twin to model infant gut ecology is, to our knowledge, unprecedented at this scale. The ability to simulate intervention outcomes, as demonstrated in our case study, positions Gi-MAPS as a powerful hypothesis-generating engine. It allows researchers and clinicians to virtually prototype and prioritize nutritional or probiotic strategies, potentially accelerating the design of more effective and personalized intervention trials (Sizemore et al., 2024; Zhu et al., 2024). This capability aligns with the growing emphasis on predictive, preventive, and personalized medicine, particularly within frameworks like China’s Healthy Child 2030 initiative.

Prior infant microbiome digital-twin studies have primarily focused on predictive correlation modeling or phenotype association using longitudinal observational data. In contrast, the Gi-MAPS CIT-Net engine is designed as an explicit ecological simulation system in which microbial abundances evolve under defined interaction parameters and functional constraints. This enables not only prediction but also mechanistic hypothesis testing through controlled *in silico* perturbations. By anchoring the digital twin in absolute microbial quantification and patent-defined engineering reproducibility, Gi-MAPS shifts digital-twin modeling from retrospective pattern fitting toward forward ecological simulation and intervention prototyping.

Gi-MAPS is designed as an open methodological framework. While initially validated in Chinese pediatric cohorts, its architecture and algorithms are adaptable for international datasets. By releasing pseudocode and synthetic data for benchmarking, Gi-MAPS enables external teams to replicate and extend its ecological-age and digital-twin models across diverse populations.

## 5 Limitations and future directions

As with any newly developed resource, Gi-MAPS has limitations that chart a clear course for future work. These limitations do not invalidate platform utility as the goal of this work is methodological standardization, not population clinical causality determination. First, while the platform’s presentation is promising, its current AI models were primarily trained and validated on Chinese pediatric cohorts. Future research must focus on external validation in diverse global populations to assess its generalizability and to calibrate ecological-age benchmarks across different genetic and environmental backgrounds.

Second, the digital-twin simulations, while insightful, represent projections based on existing data and model assumptions. Their predictive accuracy for clinical outcomes needs to be rigorously tested in prospective, randomized controlled trials. We have secured IRB approvals to expand our cohort to approximately 20,000 children, which will provide the statistical power for such validation and for refining the algorithms through continuous learning.

Third, the current version of Gi-MAPS primarily utilizes 16S rRNA and qPCR data. Future iterations will be enhanced by integrating metagenomic and metabolomic data streams. This multi-omics integration will deepen the functional insights, allowing the platform to model microbial pathways and host-interacting metabolites, thereby providing a more comprehensive systems biology view of gut health.

Fourth, a key limitation of the present study is that all AI model evaluations were performed using internal datasets, and external cohort validation remains an essential next step. While internal validation confirms engineering stability and algorithmic consistency, independent datasets from diverse populations and public microbiome repositories are required to establish generalizability and to formally exclude overfitting. Future work will benchmark Gi-MAPS against publicly available pediatric microbiome cohorts and compare its performance with existing microbiome maturity indices and digital-twin implementations.

Finally, as with all AI-driven clinical tools, issues of explainability, bias, and data security are paramount. The use of SHAP values for model interpretation is a first step. Ongoing development will need to incorporate stricter bias-detection frameworks and adhere to evolving standards for AI governance and data protection in healthcare. Future cross-validation with public datasets and integration with open-source microbiome AI frameworks (e.g., QIIME2 ML plugins) are planned to support external reproducibility.

## 6 Conclusion

In summary, Gi-MAPS establishes a new standard for integrated, AI-powered microbiome analysis with direct relevance to early-life health. By enabling strict anaerobic preservation and absolute quantification of HMO-utilizing *Bifidobacterium* species, the platform provides a quantitative foundation for assessing microbial functions that are central to infant immune maturation, gut barrier development, and metabolic programming. Gi-MAPS transforms *Bifidobacterium*-dominated early-life ecosystems from descriptive observations into measurable developmental indicators, allowing ecological immaturity and functional deficits to be detected at an individual level. We anticipate that this capability will accelerate research into the developmental origins of health and disease, support evidence-based nutritional and probiotic interventions, and ultimately enable precision strategies to guide healthy microbiome maturation in infancy and early childhood. The modular and scalable architecture of Gi-MAPS ensures it will evolve as a living resource, continuously incorporating new data and insights to refine its understanding of the nascent human gut ecosystem.

## Data Availability

All data supporting the findings of this study are contained within publicly accessible patent documents, as listed throughout the manuscript. A minimal synthetic dataset and pseudocode replicating the Gi-MAPS analytical workflow, including preprocessing, ensemble learning, co-occurrence network construction, and digital-twin simulation, are available upon justified request to the corresponding author. These materials contain no real patient data and are provided solely for reproducibility demonstration and methodological transparency.
