# Supplementary material for: Gi-MAPS: a quantitative engineering framework for AI-guided pediatric gut microbiome ecological interpretation and digital-twin simulation
Source: Front Microbiol. 2026 Mar 24;17:1739103. doi: 10.3389/fmicb.2026.1739103 (PMC13055552; doi:10.3389/fmicb.2026.1739103)
Supplement: Supplementary file 1 [file Table_1.docx]

Supplementary Table S1. Primer and probe sequences used in the multiplex qPCR assay for absolute quantification of HMO-utilizing *Bifidobacterium* species

| Target species | Forward primer (5′→3′) | Reverse primer (5′→3′) | Probe (5′→3′) |
| --- | --- | --- | --- |
| *B. breve* | AATCTGAGTGAGCGGTTGCC | GCATGACCGTCAAGTGTGGC | 5'CY5-AACGTCATCACGGCAAGGT-3'MGB |
| *B. longum* subsp. *infantis* | CGGTCTTCTACAGGAAGCGG | CCACGCTTTCCTCGTCCATA | 5'FAM-ACGACTTATGATCGATCGCG-3'MGB |
| *B. longum* subsp. *longum* | ACCAAGTTCCAGCCCACAGC | CGCCATACCAGTAGTAGGCG | 5'VIC-ACCGTGCGCTTGGATGTGT-3'MGB |
